# Cerebrovascular reactivity (PRx) and optimal cerebral perfusion pressure in elderly with traumatic brain injury

**DOI:** 10.1007/s00701-024-05956-9

**Published:** 2024-02-02

**Authors:** Samuel Lenell, Teodor Svedung Wettervik, Timothy Howells, Anders Hånell, Anders Lewén, Per Enblad

**Affiliations:** Department of Medical Sciences, Section of Neurosurgery, Uppsala University Hospital, Uppsala University, 751 85 Uppsala, Sweden

**Keywords:** Pressure reactivity index, Optimal cerebral perfusion pressure, Cerebral autoregulation, Traumatic brain injury, Elderly, Neurointensive care monitoring

## Abstract

**Purpose:**

Cerebral perfusion pressure (CPP) guidance by cerebral pressure autoregulation (CPA) status according to PRx (correlation mean arterial blood pressure (MAP) and intracranial pressure (ICP)) and optimal CPP (CPPopt = CPP with lowest PRx) is promising but little is known regarding this approach in elderly. The aim was to analyze PRx and CPPopt in elderly TBI patients.

**Methods:**

A total of 129 old (≥ 65 years) and 342 young (16–64 years) patients were studied using monitoring data for MAP and ICP. CPP, PRx, CPPopt, and ΔCPPopt (difference between actual CPP and CPPopt) were calculated. Logistic regression analyses with PRx and ΔCPPopt as explanatory variables for outcome. The combined effects of PRx/CPP and PRx/ΔCPPopt on outcome were visualized as heatmaps.

**Results:**

The elderly had higher PRx (worse CPA), higher CPPopt, and different temporal patterns. High PRx influenced outcome negatively in the elderly but less so than in younger patients. CPP close to CPPopt correlated to favorable outcome in younger, in contrast to elderly patients. Heatmap interaction analysis of PRx/ΔCPPopt in the elderly showed that the region for favorable outcome was centered around PRx 0 and ranging between both functioning and impaired CPA (PRx range − 0.5–0.5), and the center of ΔCPPopt was − 10 (range − 20–0), while in younger the center of PRx was around − 0.5 and ΔCPPopt closer to zero.

**Conclusions:**

The elderly exhibit higher PRx and CPPopt. High PRx influences outcome negatively in the elderly but less than in younger patients. The elderly do not show better outcome when CPP is close to CPPopt in contrast to younger patients.

**Supplementary Information:**

The online version contains supplementary material available at 10.1007/s00701-024-05956-9.

## Introduction

The clinical outcome after traumatic brain injury (TBI) has improved substantially with the introduction of specialized neurointensive care (NIC) [3, 4, 9, 19, 20]. The current trend in NIC treatment of TBI is towards more individualized treatment. Cerebral pressure autoregulation (CPA) status may be one important factor to consider. Promising results indicate that, instead of using fixed cerebral perfusion pressure (CPP) goals, it may be beneficial to guide CPP according to CPA status and estimated optimal CPP (the CPP range where CPA works best) [5, 25, 26, 33, 34]. CPA can be monitored by using the pressure reactivity index (PRx), which is the correlation coefficient between mean arterial blood pressure (MAP) and intracranial pressure (ICP) over 5 min [5]. Optimal CPP (CPPopt) may be calculated continuously as the CPP with the lowest PRx over a chosen period of time (hours) [1]. CPA-guided CPP was found to be safe in a recent feasibility randomized clinical trial [30] and outcome studies are under discussion.

One important fact to consider in the further development of NIC towards more individualized management is the changing demographics of TBI. The proportion of elderly (age ≥ 65) is increasing both overall and among TBI patients [8, 14, 18, 22] and is expected to increase further over time. The management of elderly TBI patients is a tremendous future challenge. The elderly differ with a higher proportion of acute subdural hematoma, higher Glasgow Coma Scale motor score (GCS M) on admission, more often exhibit chronic diseases, such as hypertension and cardiovascular disease, and are more often pre-injury treated with antithrombotic drugs [14, 15, 27, 28]. The causes of the trauma are also often different with falls in the same plane being the main cause in elderly rather than high-energy injuries [7, 10, 12–14]. Despite the known differences between elderly and younger adults with TBI, all patients are still irrespective of age treated according to the same guidelines, which are based on research predominantly on younger patients. We found in our previous study that the elderly spent more time outside the treatment thresholds, with higher CPP and higher systolic blood pressure (SBP) but seemed to benefit from this in contrast to the young adults [16]. Low SBP was found to be critical to avoid in the elderly [16]. This raises the question of whether PRx and CPPopt are useful for guidance of treatment in the elderly. Only few TBI studies have focused on CPA in the elderly [2, 6, 8]. It has been shown that cerebrovascular resistance and reactivity may change with age and that PRx appears to be better in the younger ages [24]. More studies are warranted regarding CPA specifically in elderly TBI patients.

In this study, we aimed to analyze PRx and CPPopt specifically in elderly TBI patients during NIC and relate the results to outcome. We intend to use the younger patients for comparison.

## Material and methods

### Study design and patient selection

The Department of Neurosurgery at the Uppsala University Hospital, Sweden, provides neurosurgical care for a central part of Sweden, with a population of approximately 2 million people. Most TBI patients are initially treated at local hospitals according to advanced trauma life support principles and then referred to Uppsala for NIC (the most distant hospital is 382 km away).

All patients admitted to the NIC unit have since 2008 been included in the Uppsala Traumatic Brain Injury registry [23] where patients’ characteristics, treatment characteristics, and 6-month follow-up are registered. Extended Glasgow outcome scale grade (GOSE) is assessed after around 6 months, by structured telephone interviews done by a few selected persons [31, 32].

All TBI patients admitted to Uppsala University Hospital between 2008 and 2018 aged ≥ 16 years who had available monitoring data were included in the study. Age, sex, GCS M, and GOSE were gathered from the Uppsala TBI registry. The first CT after trauma was classified according to Marshall [21], in retrospect by two of the authors (SL and TSW).

### Neurointensive care

All patients were treated according to the same local standardized treatment protocol [9]. Briefly, unconscious patients (GCS M ≤ 5) were intubated, mechanically ventilated, and had ICP monitoring regardless of age (active waiting in cases with anticoagulants/coagulopathy). Propofol was used for sedation and opiates for analgesia. An external ventricular drainage system (EVD) was used as the first choice for ICP monitoring, and an intraparenchymal pressure device was chosen in case of compressed ventricles. The pressure dome for the EVD was placed at the level of the lateral ventricles, and the arterial blood pressure dome was placed at the heart level. Moderate hyperventilation was applied initially (PaCO_2_ 4.0–4.5 kPa) and changed to normoventilation as soon as ICP permitted. Unless severe ICP elevations, regular wake-up tests were performed (3–6 times/day). Prophylactic anticonvulsants were not used. Significant mass lesions were evacuated.

The treatment goals were as follows: ICP < 20 mmHg, systolic blood pressure (SBP) > 100 mmHg, CPP > 60 mmHg, PaO_2_ > 12 kPa, glucose 5–10 mmol/L, normovolemia, electrolytes within normal ranges, and body temperature < 38 °C. PRx and CPPopt were not available bedside.

Raised ICP was treated in a stepwise fashion[9]: (1) If ICP increased ≥ 20 mmHg without mass lesions, cerebrospinal fluid (CSF) was drained. Initially (first day/days) small volumes of 1–2 ml were drained intermittently. Later when the risk for expanding hematomas and brain swelling was decreased, CSF was drained (if needed) against a pressure level of 15–20 mmHg with a continuously open EVD. (2) If ICP remained increased the treatment was escalated. No wake-up tests were performed. Patients received continuous sedation, more morphine, and stress reduction with ß1-antagonist metoprolol (0.2–0.3 mg/kg/24 h as an infusion) and α2-agonist clonidine (0.5–1.0 μg/kg × 8 or the same dose as an infusion). (3) If the ICP treatment still was insufficient, thiopental coma treatment and/or decompressive craniectomy were used as last-tier treatments. This step was initiated more restrictively in the elderly.

### Monitoring data processing

ICP and arterial monitoring data were recorded with the Odin software, developed at Uppsala University and the University of Edinburgh [11]. Collected data was screened and cleared from artifacts using the Odin software. The monitoring time left after the removal of artifacts and time gaps from, e.g., radiology examination and surgical procedures was entitled good monitoring (GMT). The proportion of GMT (% GMT) above/below certain predefined thresholds were calculated for PRx and CPPopt variables (see below).

Trended minute-by-minute data was collected for MAP, SBP, ICP, and CPP, respectively. PRx was calculated as a moving 5-min correlation of 10 s averages of ICP and MAP. PRx is presented as % GMT > 0.25. CPPopt was calculated as the CPP with the lowest PRx in the last 4 h, as described by Aries and colleagues [1]. Deviations from CPPopt were denoted ΔCPPopt and calculated as the difference between actual CPP and calculated CPPopt. ΔCPPopt is presented as % GMT with ΔCPPopt <  − 5, ± 5, or > 5 mmHg, respectively [30].

### Heatmap visualization

The combined effect of PRx/CPP and PRx/∆CPPopt, respectively, on the outcome (GOSE) was explored by creating heatmaps. The heatmaps were generated by a custom-written-R-script, developed by one of the authors (AH) as earlier described in detail [29]. The PRx range was − 1 to + 1 with a 0.05 resolution, which was combined with CPP (range 40 to 100 mmHg), and ∆CPPopt (range − 30 to + 30 mmHg), with a 2-mmHg resolution. For each coordinate/pixel (combination of two thresholds) the % GMT was calculated for all patients and correlated with GOSE using the Spearman test. Smoothing filters were used, and values were then translated into the jet color range (red to blue) with red/blue color indicating a negative/positive association with unfavorable/favorable outcomes. Coordinates/pixels with less than five patients with at least 5 min of data were colored as white. Density plots were conducted to visualize the frequency of the percentage of monitoring time for certain combinations of PRx with CPP or ∆CPPopt. The resulting numbers were normalized (divided) by the highest count within the grid to yield density values ranging from 0 to 1 for each cell in the grid. The resulting values were smoothed and then transformed into colors using the jet color scale.

### Statistics

Two age groups were analyzed, one old group ≥ 65 years of age and one young group 16–64 years old. Differences in characteristics between the age groups were analyzed with Pearson’s chi^2^ test. Non-parametric data were presented as median with interquartile range and differences between groups tested with Mann–Whitney *U*-test.

In order to analyze the influence on the outcome (favorable outcome (GOSE 5–8) and mortality) of PRx, CPPopt, and ΔCPPopt, univariate logistic regression analysis was done with favorable outcome and mortality as dependent variables. Univariate analysis was also performed for GCS M, Marshall score, and sex. Multiple logistic regression analysis was performed for favorable outcome and mortality with the explanatory variables GCS M, Marshall score, sex, % GMT with > 0.25, and % GMT with ΔCPPopt <  − 5/ ± 5/ > 5 mmHg for favorable outcome and for mortality. IBM SPSS Statistics version 28.0.1.0 was used (IBM Corp, Armonk, NY).

A *p*-value < 0.05 was considered statistically significant. In tables and figures significant findings are marked with *p < 0.05, **p < 0.01, and ***p < 0.001.

## Results

Among 471 patients who met the criteria for the study, 129 (27%) were ≥ 65 years old (old group), and 342 (73%) were between 16 and 64 years old (young group) (Table 1). The age distribution is provided as supplementary information in Online Resource 1. In the old group, 106 (82.2%) were males, the median GCS M was 5 (IQR 5–6), and the median Marshall score was 5 (IQR 2–5). In the young group, 265 (77.5%) were males, the median GCS M was 5 (IQR 4–5) and the median Marshall score was 2 (IQR 2–5).

**Table 1:** Patient characteristics

	16–64 years	≥ 65 years	*p*	
Patients, *n*	342	129		
Age (years), median (IQR)	44 (25–55)	71 (68–75)		
Sex (male), *n* (%)	265 (77.5)	106(82.2)	< 0.001^a^	***
GCS M at admission, median (IQR)	5(4–5)	5(5–6)	0.021^b^	*
Marshall score, median (IQR)	2(2–5)	5(2–5)	< 0.001^b^	***
ICP monitoring				
EVD, *n* (%)	60 (17.5)	27 (20.9)	0.398^a^	
Intraparenchymal devices, *n* (%)	196 (57.3)	84 (65.11)	0.124^a^	
Both, *n* (%)	86 (25.1)	17 (13.2)	0.005^a^	**
Neurointensive care treatment				
Craniotomy, *n* (%)	167 (48.8)	83 (64.3)	0.003^a^	**
DC, *n* (%)	44 (12.9)	6 (4.7)	0.010^a^	*
Thiopental, *n* (%)	53 (15.5)	4 (3)	< 0.001^a^	***
Outcome				
Favorable, *n* (%)	204 (59.6)	51 (39.5)	< 0.001^a^	***
Mortality, *n* (%)	37 (10.8)	40 (31.0)	< 0.001^a^	***

^a^Tested with chi^2^-test

^b^Tested with Mann–Whitney *U*-test

^*^p < 0.05, **p < 0.01, and ***p < 0.001

ICP was monitored by an EVD in 87 cases, a parenchymatous pressure device in 280, and both in 103 (Table 1). The median ICP monitoring time was 11,684 min (IQR 6672–13,491), the median MAP monitoring time was 13,081 min (IQR 8980–13,818), and the median CPP monitoring time was 11,344 (IQR 6490–13,434). The number of patients with monitoring data for each day is provided as supplementary information in Online Resource 2. Median values of each physiological parameter and median values of % GMT spent above/within/below predefined thresholds for PRx and ΔCPPopt are presented for the whole studied monitoring period (10 days) by age group in Table 2. CPPopt was possible to calculate in 53.7% of GMT in the 16–64 years group and in 56.5% of GMT in the elderly group. There were highly significant differences between the age groups. The old group showed significantly higher MAP, higher SBP, lower ICP, higher CPP, higher (worse) PRx, higher CPPopt, higher % GMT with ΔCPPopt <  − 5%, and lower % GMT with ΔCPPopt ± 5 (Table 2).

**Table 2:** Physiological features for the whole 10-day monitoring period

	16–64 years	≥ 65 years	*p*	
MAP, median (IQR)	87.1 (83.4–92.3)	91.7 (87.3–96.7)	< 0.001	***
SBP, median (IQR)	137.8 (129.9–147.4)	150.6 (140.0–158.5)	< 0.001	***
ICP, median (IQR)	12.2 (8.7–14.8)	10.6 (7.2–12.9)	< 0.001	***
CPP, median (IQR)	75.3 (70.7–81.0)	80.6 (76.1–89.1)	< 0.001	***
CPPopt, median (IQR)	75.2 (71.6–79.6)	80.8 (75.8–87.2)	< 0.001	***
PRx, median (IQR)	0.03 (− 0.06–0.12)	0.10 (0.02–0.19)	< 0.001	***
GMT PRx > 0, median % (IQR)	52.2 (42.0–63.5)	62.6 (51.2–71.8)	< 0.001	***
GMT PRx > 0.25, median % (IQR)	26.9 (20.4–37.2)	36.6 (26.8–46.0)	< 0.001	***
GMT PRx > 0.35, median % (IQR)	19.5 (14.2–27.9)	26.0 (18.4–35.3)	< 0.001	***
GMT ΔCPPopt < − 5, median % (IQR)	30.9 (23.0–40.2)	34.9 (25.7–44.0)	0.014	*
GMT ΔCPPopt ± 5, median % (IQR)	28.4 (23.4–34.2)	24.4 (20.5–27.9)	< 0.001	***
GMT ΔCPPopt > 5, median % (IQR)	33.1 (25.2–40.5)	33.9 (25.6–43.8)	0.236	

Difference between age groups tested with Mann–Whitney *U*-test

^*^p < 0.05, **p < 0.01, and ***p < 0.001

**Fig. 1:**
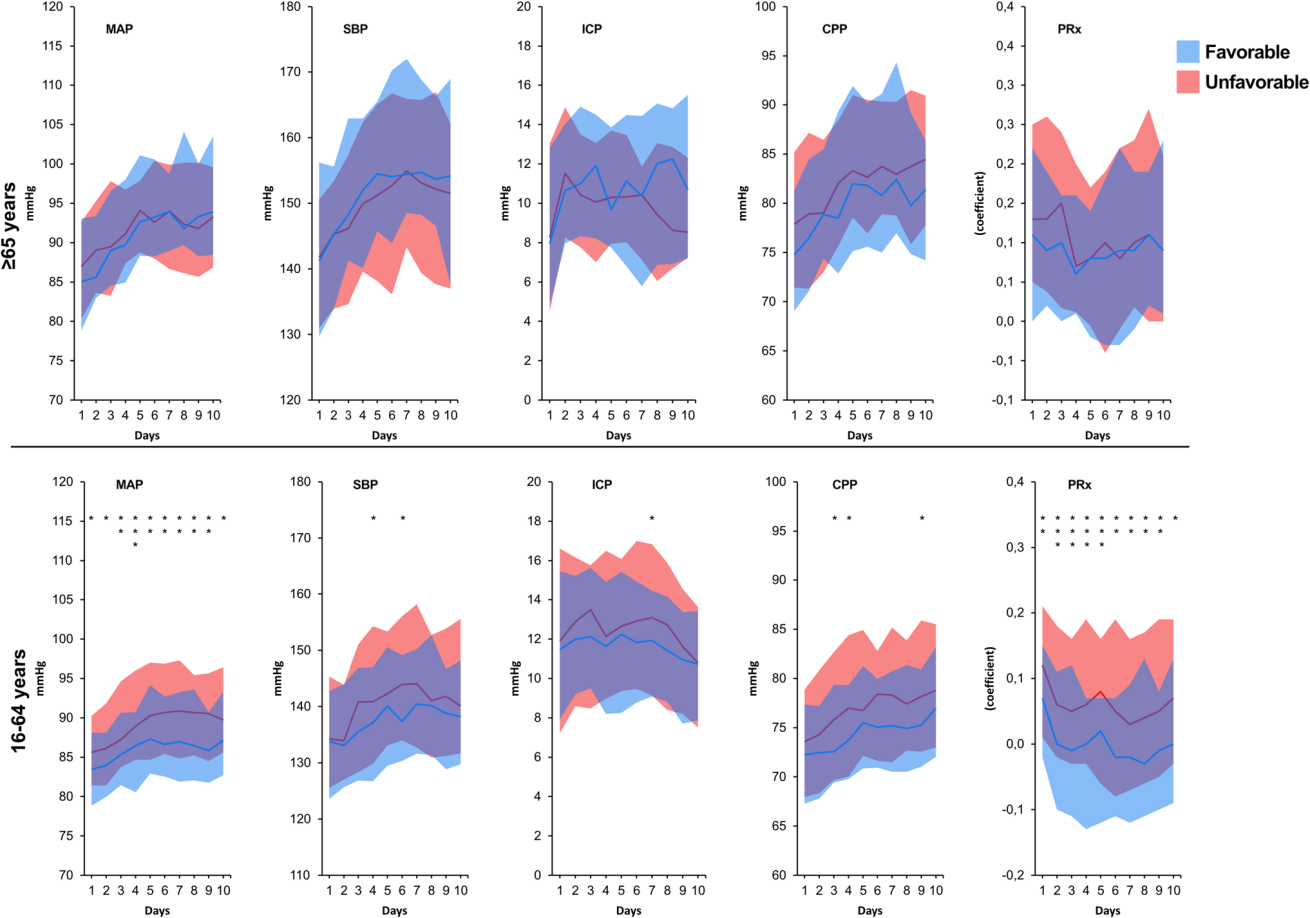
Temporal daily distribution of MAP, SBP, ICP, CPP, and PRx by outcome and age group. Distribution of patients’ daily mean values on group level for each physiological feature with the distribution presented as median (line) and IQR (band). Favorable outcome GOSE 5–8 and unfavorable GOSE 1–4. Difference between favorable and unfavorable tested for each day with Mann–Whitney *U*-test. *p < 0.05, **p < 0.01, and ***p < 0.001

**Fig. 2:**
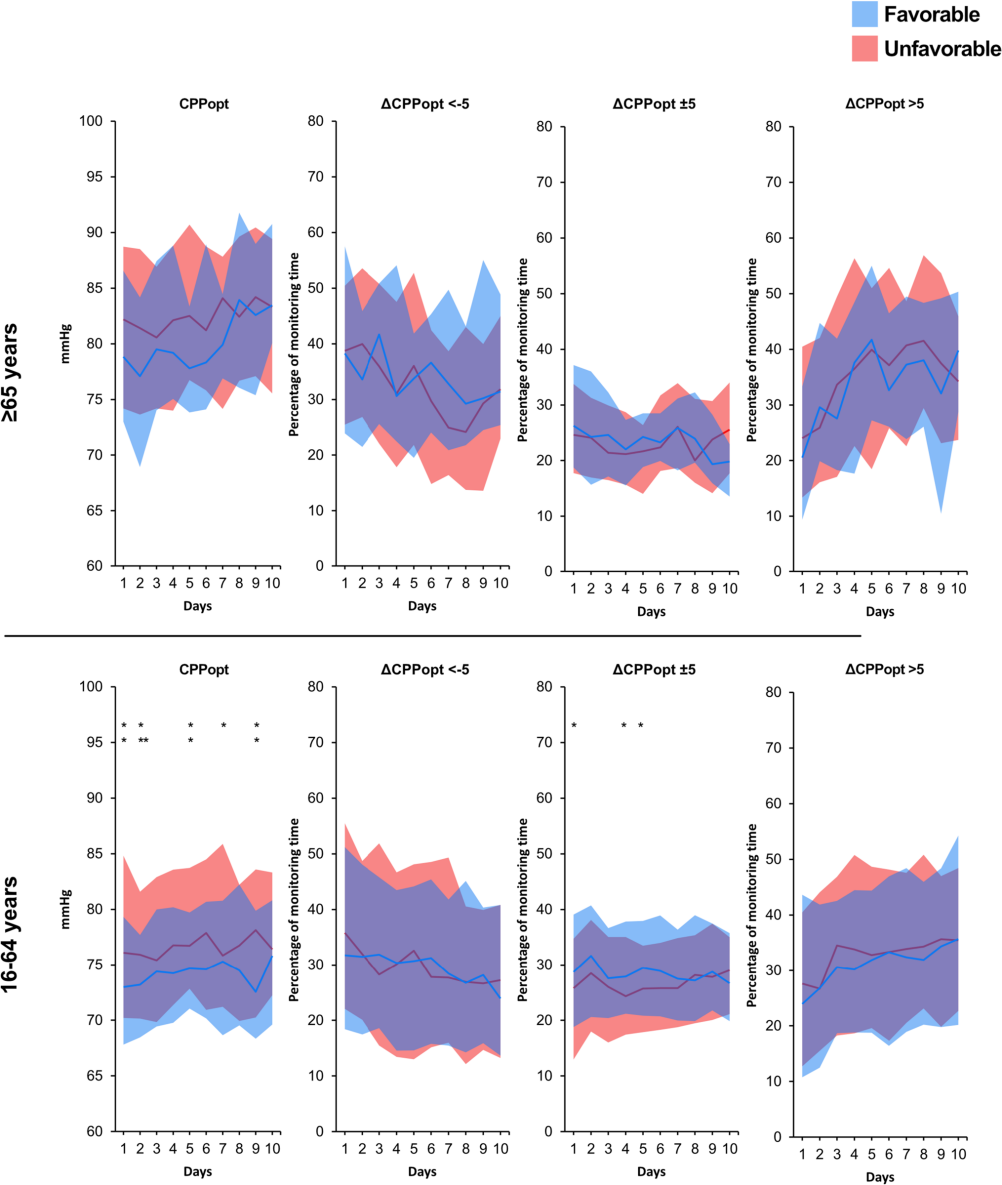
Temporal daily distribution of CPPopt and ΔCPPopt by outcome and age group. Distribution of patients’ daily mean values on group level for CPPopt and mean percentage monitoring time of ΔCPPopt <  − 5, ΔCPPopt ± 5, and ΔCPPopt > 5 over 10 days with the distribution presented as median (line) and IQR (band). Favorable outcome GOSE 5–8 and unfavorable GOSE 1–4. Difference between favorable and unfavorable tested for each day with Mann–Whitney *U*-test. *p < 0.05, **p < 0.01, and ***p < 0.001

When the temporal patterns of MAP, SBP, ICP, CPP, and PRx were analyzed in the old group by day and divided into favorable outcome and unfavorable outcomes, it seemed to be a tendency that patients ≥ 65 years with lower MAP days 8–10, lower SBP days 3–10, and higher PRx days 0–5 had more unfavorable outcome (Fig. 1). In the young group, patients with higher PRx and higher MAP had significantly more unfavorable outcome during almost the whole time period (Fig. 1). Analysis of temporal patterns of CPPopt and % GMT with ΔCPPopt <  − 5/ ± 5/ > 5 showed no significant differences between favorable and unfavorable outcomes at any day in the old group (Fig. 2). In the young group, patients with unfavorable outcome had significantly higher median of CPPopt almost all days (days 1, 2, 5, 7, and 9) and significantly lower % GMT with ΔCPPopt ± 5 days 1, 4, and 5 (Fig. 2).

The logistic regression analyses for the whole period with favorable outcomes and mortality as dependent variables are presented in Table 3. In the young group, both the univariate and the multivariate analyses showed significantly lower odds ratio (OR)/adjusted odds ratio (AOR) for favorable outcome with increasing Marshall score and increasing % GMT with PRx > 0.25, and significantly higher OR/AOR for favorable outcome with increasing GCS M. Higher ΔCPPopt ± 5 showed significantly higher OR for favorable outcome in the univariate analysis but not in the multivariate analysis. None of the variables showed statistical significance in the elderly group for favorable outcomes, neither in the univariate nor in the multiple regression analysis. In the univariate regression analysis for mortality, the young group had significantly higher OR for mortality with increasing Marshall score, increasing % GMT with PRx > 0.25, and increasing % GMT with ΔCPPopt <  − 5 (Table 3). Higher GCS M and higher % GMT with CPPopt ± 5 were significantly associated with lower OR for mortality. In the multiple regression analysis, the young group had a significantly higher AOR for mortality with PRx > 0.25 and a significantly lower AOR with higher GCS M on admission (Table 3). In the old group, significantly higher OR/AOR for mortality was seen for a higher % GMT with PRx > 0.25 in both the univariate and multiple logistic regression analyses but no other significant associations were found (Table 3).

**Table 3:** Logistic regression analysis (whole 10 days period) with favorable and mortality as dependent

Variable	16–64 years							
	Univariate logistic regression				Multiple logistic regression			
	OR	95% CI	*p*		AOR	95% CI	*p*	
Favorable model^c^								
Sex (male)	1.005	0.599–1.686	0.985		1.134	0.628–2.046	0.676	
GCS M on admission	1.852	1.499–2.288	< 0.001	***	1.914	1.484–2.467	< 0.001	***
Marshall score	0.763	0.655–0.889	< 0.001	***	0.809	0.679–0.964	0.018	*
PRX > 0.25 (%GMT)	0.964	0.948–0.98	< 0.001	***	0.973	0.953–0.993	0.009	**
ΔCPPopt < − 5 (%GMT)	0.995	0.979–1.011	0.520		1.211	0.854–1.718	0.282	
ΔCPPopt ± 5 (%GMT)	1.048	1.016–1.082	0.003	**	1.287	0.866–1.912	0.213	
ΔCPPopt > 5 (%GMT)	0.987	0.968–1.005	0.164		1.184	0.835–1.679	0.343	
Mortality model^d^								
Sex (male)	0.783	0.33–1.86	0.580		0.830	0.292–2.358	0.726	
GCS M on admission	0.648	0.514–0.818	< 0.001	*	0.784	0.562–0.995	0.046	*
Marshall score	1.313	1.048–1.645	0.018	*	1.243	0.949–1.627	0.114	
PRX > 0.25 (%GMT)	1.057	1.035–1.08	< 0.001	***	1.029	1.000–1.058	0.046	*
ΔCPPopt < − 5 (%GMT)	1.040	1.013–1.067	0.003	**	1.118	0.715–1.750	0.625	
ΔCPPopt ± 5 (%GMT)	0.915	0.865–0.969	0.002	**	1.066	0.643–1.768	0.803	
ΔCPPopt > 5 (%GMT)	0.980	0.949–1.012	0.221		1.107	0.707–1.733	0.658	

Variable	≥ 65 years							
	Univariate logistic regression				Multiple logistic regression			
	OR	95% CI	*p*		AOR	95% CI	*p*	
Favorable model^c^								
Sex (male)	0.616	0.234–1.624	0.328		0.858	0.302–2.439	0.774	
GCS M on admission	1.186	0.864–1.629	0.292		1.158	0.823–1.630	0.401	
Marshall score	0.949	0.763–1.18	0.636		0.958	0.758–1.212	0.721	
PRX > 0.25 (%GMT)	0.983	0.96–1.006	0.148		0.986	0.960–1.013	0.300	
ΔCPPopt < − 5 (%GMT)	1.001	0.978–1.025	0.932		0.905	0.535–1.533	0.711	
ΔCPPopt ± 5 (%GMT)	1.024	0.972–1.079	0.364		0.907	0.496–1.658	0.751	
ΔCPPopt > 5 (%GMT)	0.991	0.965–1.018	0.502		0.897	0.529–1.520	0.685	
Mortality model^d^								
Sex (male)	0.968	0.364–2.575	0.948		1.084	0.357–3.292	0.887	
GCS M on admission	0.822	0.605–1.115	0.208		0.879	0.616–1.254	0.477	
Marshall score	1.196	0.944–1.515	0.137		1.159	0.888–1.513	0.278	
PRX > 0.25 (%GMT)	1.044	1.017–1.071	0.001	**	1.035	1.005–1.066	0.023	*
ΔCPPopt < − 5 (%GMT)	1.015	0.991–1.041	0.227		1.117	0.637.1.961	0.699	
ΔCPPopt ± 5 (%GMT)	0.983	0.93–1.038	0.535		1.127	0.594–2.140	0.714	
ΔCPPopt > 5 (%GMT)	0.986	0.959–1.015	0.343		1.108	0.630–1.947	0.722	

Univariate logistic regression analyses for variables from each age group with favorable or mortality as dependent. For each age group multiple regression analyses were made for favorable outcome and mortality taking all variables into account. *p < 0.05, **p < 0.01, and ***p < 0.001

^c^16–64 years: Nagelkerke R square = 0.254. ≥ 65 years; Nagelkerke R square = 0.049

^d^16–64 years: Nagelkerke R Square = 0.176. ≥ 65 years; Nagelkerke R square = 0.135

**Fig. 3:**
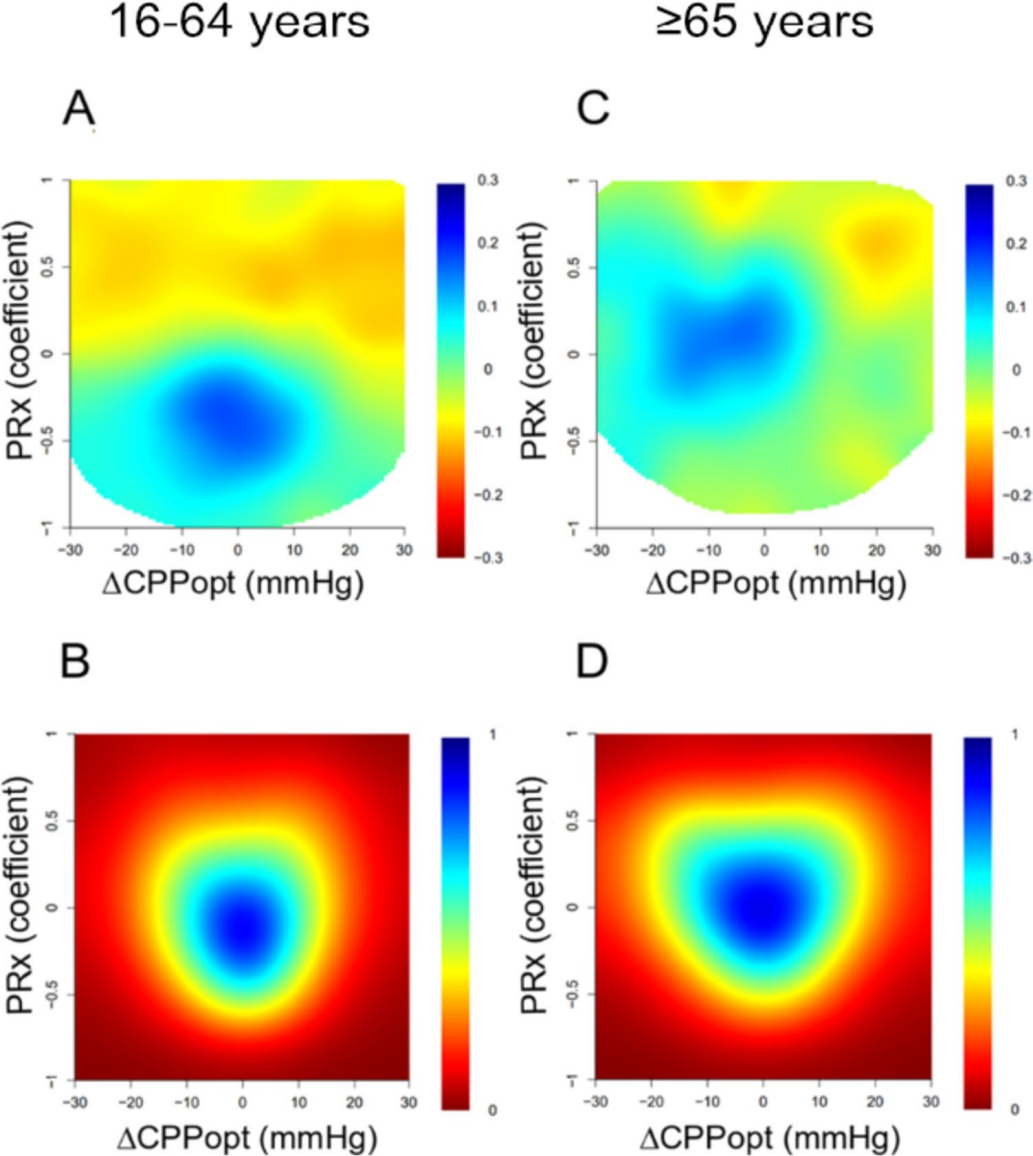
Combined effect of PRx and ΔCPPopt on clinical outcome. The figure illustrates the combined association of the percentage of monitoring time (% GMT) for absolute PRx and ∆CPPopt values with GOSE (**A** and **C**) and density plots with the data frequency of certain PRx and ∆CPPopt combinations (**B** and **D**). The % GMT for the concurrent combination of PRx and ∆CPPopt during the 10 days was calculated and correlated with GOSE. The jet color range denotes the value of the correlation coefficients, where blue color indicates favorable and red color indicates unfavorable outcome. Pixels with less than five patients with 5 min of monitoring with a certain combination of PRx and CPP were colored as white

**Fig. 4:**
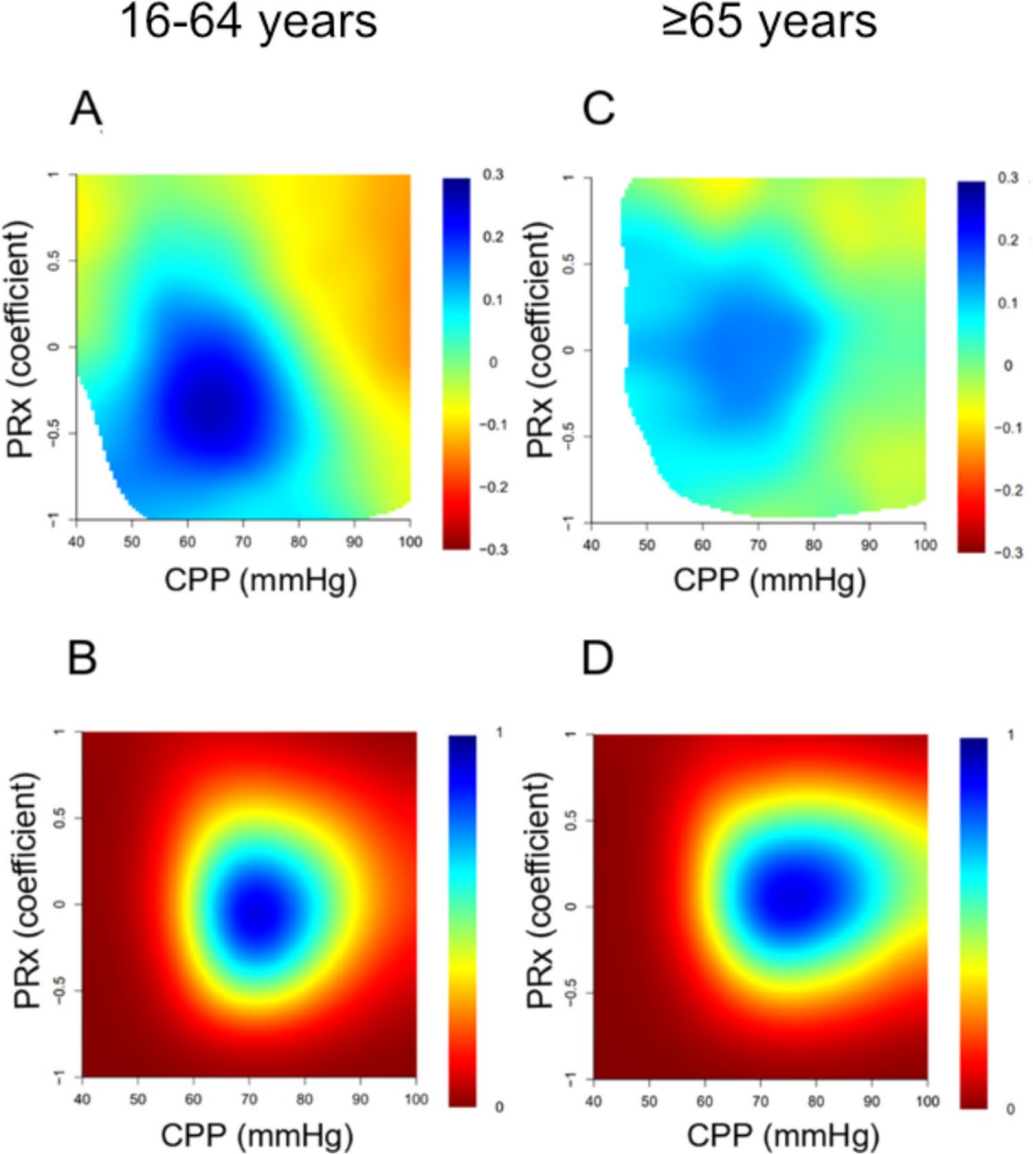
Combined effect of PRx and CPP on clinical outcome. The figure illustrates the combined association of the percentage of monitoring time (% GMT) for absolute PRx and CPP values with GOSE (**A** and **C**) and density plots with the data frequency of certain PRx and CPP combinations (**B** and **D**). The % GMT for the concurrent combination of PRx and CPP during the 10 days was calculated and correlated with GOSE. The jet color range denotes the value of the correlation coefficients, where blue color indicates favorable and red color indicates unfavorable outcome. Pixels with less than five patients with 5 min of monitoring with a certain combination of PRx and CPP were colored as white

Heatmap interaction analysis of PRx/ΔCPPopt in the elderly showed that the field for favorable outcome had its center around PRx 0 (range − 0.5–0.5) and ΔCPPopt − 10 (range − 20–0) and that the plots were more dispersed than in the younger patients who had a center for favorable outcome around PRx − 0.5 (range − 0.75–0) and ΔCPPopt closer to zero (range − 10–10 (Fig. 3). The density plots showed almost the same center in both age groups (marginally lower PRx center in the younger) but with a wider field in the elderly group (Fig. 3). In the PRx/CPP interaction heatmap, the elderly showed a more dispersed field for favorable outcome compared to the young group (Fig. 4). In the old group, the field of favorable outcome mostly fitted in between PRx − 0.5 and 0.5 and CPP between 60 and 80 in contrast to the younger group where the field had a more distinct center at approximately PRx − 0.3 (range PRx − 0.7–0.4) and CPP 65 (range CPP 50–80). The density plots had approximately the same center of PRx in both groups, but the elderly group has more values in the higher CPP range than the young (Fig. 4).

## Discussion

In this single-center study, we analyzed monitoring data from 129 elderly TBI patients (≥ 65 years) and compared the results with 342 younger TBI patients (16–64 years), with particular interest in CPA. All patients were treated during the same period according to the same protocol. The cerebrovascular indices were calculated in retrospect in order to evaluate the potential of using PRx and CPPopt for guidance of CPP treatment in the elderly. Our concern was that older patients may differ, especially since we found in our previous study that the elderly spent more time with higher CPP and higher systolic blood pressure (SBP) but seemed to benefit from this in contrast to the young adults [16].

In this study, the elderly proved to have higher median values of PRx, which is in accordance with the findings by Czonyka et al. [6]. We observed also that the median CPPopt was higher in the elderly. Furthermore, the elderly spent a higher % GMT with higher PRx values and a higher % GMT with CPP outside ΔCPPopt ± 5 (Table 2). These findings were also consistent with the density plots (Fig. 3). It appears convincing that elderly TBI patients have worse CPA and spend less time where the CPA works best in comparison to younger patients. The reasons for the age differences probably depend on multiple factors, e.g., different dominating types of brain injury, co-existing cardio- and cerebrovascular diseases, and medication. In order to evaluate the potential of using PRx and CPPopt for individualized treatment of CPP in the elderly, analysis of the impact on outcome may give insights.

When the temporal patterns during the whole study period of 10 days were analyzed for the monitoring parameters by outcome (Fig. 1), old patients with unfavorable outcomes tended to have lower MAP days 8–10, lower SBP days 3–10, and higher PRx days 0–5. A different picture was found for the young group where PRx and MAP were significantly higher in patients with unfavorable outcomes during the whole study period. Looking at mean CPPopt and ΔCPPopt, no significant correlations with outcome were found in the elderly (Fig. 2). In the young group on the other hand, high mean CPPopt was significantly related to worse outcome half of the days (days 1, 2, 5, 7, and 9) and high % GMT with ΔCPPopt ± 5 was significantly related to favorable outcome (day 1, 4, and 5). The overall impression was thus that median CPP and proportion of time with CPP close to CPPopt, above CPPopt, or below CPPopt exert a greater impact on outcome in patients who are young and that those factors are less important in the elderly.

Looking at the logistic regression analysis of the whole monitoring period, poor cerebrovascular reactivity (high PRx) proved consistently to be associated with unfavorable outcome and mortality in the young group, both in the univariate and multivariate analyses (Table 3). Furthermore, in the young group large % GMT with ΔCPPopt ± 5 was significantly related to favorable outcome in the univariate analysis although no independent influence on outcome was found in the multivariate analysis. Regarding mortality, large % GMT with ΔCPPopt <  − 5 and small % GMT with ΔCPPopt ± 5 were significantly associated to mortality in the univariate analysis of the young group, although no significant associations were found in the multivariate analysis. In the old group, the only significant finding was that CPA was associated to mortality both in the univariate and multivariate analyses Detailed interpretation of the differences found between the young and old groups is difficult but may probably to some extent be explained by the observed differences in physiological monitoring features. However, the results indicate that cerebrovascular reactivity and deviations from CPPopt play a more important role for the clinical course in younger patients than in the elderly.

Another way of studying the significance of CPA and deviations from CPPopt is to visualize the interactions of PRx with CPP and ∆CPPopt, respectively, by generation of heatmaps. The heatmaps also indicated that cerebrovascular reactivity and small ΔCPPopt are more important in the young group. In the PRx/CPPopt heatmap, the elderly showed that the field for favorable outcome had its center around PRx 0 and was ranging between both functioning and impaired CPA (PRx range − 0.5–0.5) and that the center of ΔCPPopt was at − 10 (ranging between − 20 and 0), and the plots were more dispersed than in the younger patients who had the center for favorable outcome at around PRx − 0.5 with a field within functioning CPA (PRx range − 0.75–0) and the center of ΔCPPopt closer to zero (range − 10–10 (Fig. 3).

More studies of CPA in the elderly are warranted to substantiate our findings. Many questions remain to be answered that require multicenter studies with a large number of elderly patients, e.g., the impact of injury type and cardiovascular status. Careful consideration is always needed before the implementation of new treatment strategies, and we believe our results highlight that management principles that originate from younger TBI patients cannot be directly generalized to the elderly. Hence, before introducing CPA-guided CPP management in the elderly, more knowledge regarding CPA must be gathered from observational studies. The introduction of non-standardized CPA-guided management should be avoided in order not to bias the observational studies. At present our findings only indicate that it may be beneficial with relatively high blood pressure and high CPP in the elderly.

There are some limitations of the study that need to be considered. The study was retrospective, although data were prospectively collected. The results must be validated in other centers since this was a single-center study, and generalization of the results to other centers needs to be done with caution. It should also be mentioned that there is a referral selection bias, especially for the elderly since patients with more severe injuries and/or significant comorbidity considered not possible to treat were not accepted. The effect of a treatment bias must also be considered. The policy was that thiopental coma treatment and/or decompressive craniectomy should be initiated more restrictively in the elderly. This was also true in reality. The selection bias and treatment bias may have influenced the results, but these circumstances are what we have to deal with in reality. Furthermore, there were multiple comparisons but since this was an observational study we did not adjust for that. The fact that CPPopt was only possible to calculate in slightly above 50% of the GMT is a weakness of the concept, although this finding did not differ substantially between the age groups. Using the multi-window method described by Liu and colleagues [17] may have improved the CPPopt yield but since most earlier studies of CPPopt are based on the original 4-h window we preferred to use that.

In conclusion, the results of this study show that the elderly have higher PRx (worse autoregulation) and higher CPPopt; that high PRx influences outcome negatively in elderly patients but to a lesser extent than in the younger patients; and that more time spent close to CPPopt is associated with favorable outcome in younger patients but not in the elderly. Thus, CPA-guided therapy seems less promising in the elderly. Accordingly, the differences found for the elderly need to be considered when studies of CPPopt-guided therapy are designed since the inclusion of elderly patients may confound the results, and power analysis may be misled.

## Supplementary Information

Below is the link to the electronic supplementary material.

Supplementary file1 (DOCX 16.3 kb)

Supplementary file2 (DOCX 15.4 kb)

## Abbreviations

CPP: Cerebral perfusion pressure
CPA: Cerebral pressure autoregulation
PRx: Pressure reactivity index
MAP: Mean arterial pressure
ICP: Intracranial pressure
CPPopt: Optimal CPP
TBI: Traumatic brain injury
ΔCPPopt: Difference between CPP and CPPopt
NIC: Neurointensive care
GCS M: Glasgow Coma Scale motor score
SBP: Systolic blood pressure
GOSE: Extended Glasgow outcome scale
EVD: External ventricular drainage system
CSF: Cerebrospinal fluid
GMT: Good monitoring time
% GMT: Proportion of GMT
IQR: Interquartile range
OR: Odds ratio
AOR: Adjusted odds ratio
DC: Decompressive craniectomy
CI: Confidence interval
