# Supplementary material for: Cerebrovascular reactivity (PRx) and optimal cerebral perfusion pressure in elderly with traumatic brain injury
Source: Acta Neurochir (Wien). 2024 Feb 2;166(1):62. doi: 10.1007/s00701-024-05956-9 (PMC10837240; doi:10.1007/s00701-024-05956-9)
Supplement: Supplementary file 1 — Supplementary file1 (DOCX 16.3 kb) [file 701_2024_5956_MOESM1_ESM.docx]

**Online Resource 1.** Age distribution
