# Supplementary material for: Cerebrovascular reactivity (PRx) and optimal cerebral perfusion pressure in elderly with traumatic brain injury
Source: Acta Neurochir (Wien). 2024 Feb 2;166(1):62. doi: 10.1007/s00701-024-05956-9 (PMC10837240; doi:10.1007/s00701-024-05956-9)
Supplement: Supplementary file 2 — Supplementary file2 (DOCX 15.4 kb) [file 701_2024_5956_MOESM2_ESM.docx]

**Online Resource 2** Number of patients with monitoring data for each physiological feature and age group by day.

| Day |  | 1 | 2 | 3 | 4 | 5 | 6 | 7 | 8 | 9 | 10 |
| --- | --- | --- | --- | --- | --- | --- | --- | --- | --- | --- | --- |
| MAP, n (%) | 16-64 years | 329 (96.2) | 326 (95.3) | 317 (92.7) | 310 (90.6) | 297 (86.8) | 274 (80.1) | 268 (78.4) | 254 (74.3) | 234 (68.4) | 211 (61.7) |
|  | ≥65 years | 127 (98.4) | 124 (96.1) | 121 (93.8) | 114 (88.4) | 108 (83.7) | 98 (76.0) | 92 (71.3) | 81 (62.8) | 77 (59.7) | 69 (53.5) |
| BPs, n (%) | 16-64 years | 329 (96.2) | 326 (95.3) | 318 (93.0) | 310 (90.6) | 297 (86.8) | 274 (80.1) | 268 (78.4) | 254 (74.3) | 234 (68.4) | 211 (61.7) |
|  | ≥65 years | 127 (98.4) | 124 (96.1) | 121 (93.8) | 114 (88.4) | 108 (83.7) | 98 (76.0) | 92 (71.3) | 81 (62.8) | 77 (59.7) | 69 (53.5) |
| ICP, n (%) | 16-64 years | 306 (89.5) | 314 (91.8) | 307 (89.8) | 298 (87.1) | 288 (84.2) | 263 (76.9) | 243 (71.1) | 231 (67.5) | 212 (62.0) | 192 (56.1) |
|  | ≥65years | 112 (86.8) | 115 (89.1) | 114 (88.4) | 103 (79.8) | 93 (72.1) | 87 (67.4) | 74 (57.4) | 63 (48.8) | 57 (44.2) | 47 (36.4) |
| CPP, n (%) | 16-64 years | 307 (89.8) | 313 (91.5) | 306 (89.5) | 296 (86.5) | 285 (83.3) | 260 (76.0) | 242 (70.8) | 227 (66.4) | 209 (61.1) | 188 (55.0) |
|  | ≥65 years | 112 (86.8) | 114 (88.4) | 113 (87.6) | 102 (79.1) | 93 (72.1) | 86 (66.7) | 74 (57.4) | 62 (48.1) | 57 (44.2) | 47 (36.4) |
| CPPopt, n (%) | 16-64 years | 287 (83.9) | 306 (89.5) | 297 (86.8) | 290 (84.8) | 275 (80.4) | 254 (74.3) | 237 (69.3) | 224 (65.5) | 201 (58.8) | 181 (52.9) |
|  | ≥65 years | 103 (79.8) | 108 (83.7) | 109 (84.5) | 99 (76.7) | 88 (68.2) | 83(64.3) | 71 (55.0) | 61 (47.3) | 55 (42.6) | 47 (36.4) |
| PRx, n (%) | 16-64 years | 301 (88.0) | 310 (90.6) | 302 (88.3) | 291 (85.1) | 279 (81.6) | 256 (74.9) | 237 (69.3) | 223 (65.2) | 204 (59.6) | 181 (52.9) |
|  | ≥65 years | 109 (84.5) | 112 (86.8) | 111 (86.0) | 99 (76.7) | 89 (69.0) | 83 (64.3) | 72 (55.8) | 61 (47.3) | 57 (44.2) | 47 (36.4) |
